# The Association between Glucose Control and Functional Indices in Older People with Diabetes

**DOI:** 10.1155/2018/1053815

**Published:** 2018-12-23

**Authors:** M. Azmon, N. Gayus, H. Michal, L. Olmer, T. Cukierman-Yaffe

**Affiliations:** ^1^Physiotherapy Department, Ariel University, Ariel, Israel; ^2^The “Segal” Center for Successful Aging with Diabetes, Endocrinology Institute, Sheba Medical Center, Israel; ^3^The Epidemiology Department, Sackler School of Medicine, Tel-Aviv University, Israel

## Abstract

Diabetes may be viewed as a disease of accelerated aging as it is a risk factor for physical disability and impairment in simple and complex activities, and is a higher risk for falls and fractures. Data from the last several years suggests that this increased risk is due not only to recognized diabetes complications but also to an accelerated decline in physical capacity due to lower muscle quality and a more rapid decline in muscle mass and lower extremity strength over time. *Aim*. To find the association between glucose control and functional indices. *Methods*. A cross-sectional study conducted at the Center for Successful Aging with Diabetes at the Sheba Medical Center. Individuals with a diagnosis of type 2 diabetes over the age of 60 were included. Functional status was assessed using tools that assess aerobic, strength, and balance capacities. Medical assessment was conducted through interview, physical examination, and collection of information from medical records. The association between functional indices and A1C was assessed using linear regression. *Results*. 153 consecutive individuals were evaluated. There was an inverse association between A1C and score achieved on the 6-minute walk; with increasing meters walked on the 6-minute walk test, there was a reduction in A1C (*p* = 0.003). There was also an inverse association with the 10-meter walk (*p* = 0.007), BERG balance test (*p* = 0.0006), and timed up and go (*p* = 0.01). *Conclusion*. In this cohort of older people with diabetes, there was an association between A1C and measures of functional indices. Future studies of glucose-lowering medication should include physical and functional assessment.

## 1. Introduction

The population is aging. Thus, it is expected that by the year 2050 more than 25% of the world's population will be over the age of 60 [1]. Increasing life expectancy is associated with increased morbidity and more years spent in prolonged disability and dependency. The prevalence of diabetes is high in the elderly population. In the US it has been reported that 25–33% of the population over the age of 65 have diabetes [2]. In Israel, according to the Quality Indicators Report 2012-2014, 28% of the 65-74 age group rising to 32% of the 75-84 age group have diabetes. Diabetes is a disease that accelerates processes of aging [3].

Disability denotes the presence of “limitation in performance of socially defined roles and tasks within a sociocultural and physical environment” [4]. Frailty and sarcopenia are both risk factors for the development of disability. Frailty is a clinical entity characterized by decreased reserve and resistance to stressors resulting from declines in many physiological systems [5]. Sarcopenia is an important cause of frailty in older people. Sarcopenia is defined as low muscle mass together with low muscle function (strength or performance) [6].

People with diabetes have an increased risk of disability, have more impairment in simple and complex activities of daily living then those without diabetes after controlling for age [7, 8], and also have a higher risk for falls and fractures. In a systematic review of 26 studies with sample sizes between 369 and 66813, diabetes increased the risk for mobility disability by 1.5 (95% CI 1.38, 1.64), instrumental daily activity (IADL) disability by 1.6 (95% CI 1.55, 1.74), and activities of daily living (ADL) disability by 1.8 (95% 1.40, 2.36) [8].

There are many suggested etiologies for disability among adults with diabetes such as vision impairment, cardiovascular morbidity, peripheral neuropathy, and kidney failure. Data from the last several years suggests that people with diabetes also have an accelerated loss of muscle mass, strength, and function (i.e., sarcopenia). Indeed, it has been suggested that sarcopenia is an intermediate step in the development of frailty and disability in older people with diabetes [5, 6, 9, 10]. It is therefore important to try and identify risk factors for developing sarcopenia in older people with diabetes, especially if they are modifiable.

Thus, *the purpose* of this study is to assess the association between the glucose levels as measured by A1C and physical abilities in people over the age of 60 with diabetes.

## 2. Research Design and Methods

This was a cross-sectional study conducted at the Center for Successful Aging with Diabetes at the Sheba Medical Center. 153 Hebrew-speaking individuals with a diagnosis of type 2 diabetes over the age of 60 were included. People were either self-referred or referred by treating physician due to difficulties in managing their disease. Excluded were people with significant visual, hearing, motor, or cognitive impairment that may have precluded neuropsychological testing and responding to self-report questionnaires. Physical status was assessed using tools that assess aerobic, strength, and balance capacities utilizing the timed up and go [11–14], 6-minute walk test [11, 15, 16], 10-meter walk [17–19], Berg balance scale (BBS) [20], four step square test (FSST) [21], 30-second sit to stand [22], grip strength using a Jamar dynamometer [23–26], Fried criteria for frailty [5], and Baecke physical activity questionnaire [27]. Medical assessment was conducted through interview, physical examination, and collection of information from medical records. Data pertaining to weight, hypertension, smoking status, dyslipidemia status (lipid profile conducted routinely every several months in people with diabetes), glucose control, diabetes duration, and diabetes complications (retinopathy, nephropathy, neuropathy, cerebrovascular disease, cardiovascular disease, peripheral vascular disease) was collected. For a more elaborate description of the procedures conducted during the evaluation day, see Reference [28].

### 2.1. Measurements

There are many physical indices that can be used to assess physical and functional performance. Among them are the short physical performance battery (SPPB) and the 400-meter walk test [27]. The following physical indices were chosen as they pertain to important physical domains, are widely used and well validated, and are widely distributed in a relatively physically intact population.

#### 2.1.1. Gait, Balance, and Aerobic Capacity Measurements

The following instruments were used to assess gait speed balance and aerobic capacity by the physiotherapist.

### 2.2. Timed Up and Go [11–14]

The objective of this test is to measure the ability of a person to stand up, walk, turn around, and sit down safely in a timely manner. The test examines most mobility skills. The participant is told to get up from a chair with handles, walk 3 meters, turn, walk back, and sit down again. The score is according to the length of time in seconds to complete the task. The score is categorized according to the risk for falls and independent walking. The following cut-offs are conventionally used: less than14 seconds = independent mobility; 15-20 seconds = semi-independent mobility, may have a somewhat increased risk for falls and needs further evaluation, and some may need a walking aid; and 20-30 seconds = dependent mobility: need help walking, 50% with a cane, 40% walker, and 10% supervision. Some will need help in transfers, and most will require help using the toilet. Many in this category will not go outside the home alone.

Data suggests that the timed “up and go” test is a reliable and valid test for quantifying functional mobility that may also be useful in following clinical change over time.

### 2.3. The 6-Minute Walk Test [11, 15, 16]

The 6-minute walk test (6MWT) measures the distance an individual is able to walk over a total of six minutes on a hard, flat surface. The goal is for the individual to walk as far as possible in six minutes. The individual is allowed to self-pace and rest as needed as they traverse back and forth along a marked walkway. The 6-minute walk distance in healthy adults has been reported to range from 400 m to 700 m. People with lower vs. higher scores on the 6-minute walk are at higher risk for falls, disability, frailty, hospitalization, and death.

### 2.4. 10-Meter Walk Test [17–19]

The test examines the pace and number of steps it takes a person to pass 10 meters. A route of 10 meters is marked by two lines, and a chair is placed two meters past the runway end line. The subject starts the test two meters before the runway and goes 14 meters (two meters for acceleration at the beginning and two meters for deceleration at the end). The score achieved is determined by the time lapsed by the participant during walking along the middle 10 meters. Subject performs the test four times; the first two times are for practice: measurement occurs only during the third and fourth times. In addition to measuring the speed, the number of steps required to cross the short distance is also counted. Studies have a shown that better gait speed is associated with a lower risk for functional decline, hospitalization, and mortality [18, 19].

### 2.5. Berg Balance Scale (BBS) [20]

The Berg balance test includes 14 tasks which evaluate static and dynamic balance. Each task receives a score of 0 to 4 points—depending on the quality and task execution time 40-42. The maximum score is 56 points. The scores are dichotomized in the following manner:

(1) Scores below 36 indicate impairment with an increased risk for falls
(2) Scores between 37 and 45 indicate need for a walking aid in order to walk in a safe manner
(3) Scores above 45 indicate an independent walker without an increased risk of falls
(4) The equipment used for the Berg balance test is a step stool, a mat table, a chair with arms, a tape measure, a stopwatch, a pen, and a table. Studies have shown that individuals with scores indicating impaired balance are at increased risk of falls resulting in hospitalizations and deaths [20].

### 2.6. Four Square Step Test (FSST) [21]

The test evaluates dynamic balance in a high functional level and features walking forward, backward, left, and right above 2. 90 cm and 2.5 cm high long sticks divide the floor into four squares. The participant stands in square 1 facing the no. 2 square. The goal is to walk as quickly as possible in all the squares in the following order: from 1 to 2, 3, 4, 1, 4, 3, 2, and 1 without touching the sticks. The score is the time required to complete the entire route.

### 2.7. The 30 sec Sit to Stand Test [22]

This test examines the strength of the lower extremities. 30-second chair stand: its purpose is to evaluate the strength of the lower extremities. The instructions for the subject are to stand up for a full session as many times as he can, without the help or push of the hands (his hands crossed on his chest) for 30 seconds. The score is determined by the number of times the subject is able to achieve full compliance [22]. It was found that this test can discriminate between age-related physical decline without increased risk of falls and accelerated decline associated with increased risk of falling.

### 2.8. Grip Strength Test for Upper Limb Force Assessment [23–26]

The maximum grip strength test is measured using the Jamar dynamometer. The score is the average in kilograms. This score is compared to the general population according to age and sex [25]. Studies show that the grip decreases after midlife. It has been shown that the holding strength at old age has predictability: low scores have a relationship to falls, disability, health-related quality of life, longer hospitalization, and death. The strength test is an effective test to assess the degree of aging, nutrition, and overall condition of the subject [26].

### 2.9. Additional Assessment

#### 2.9.1. Frailty [5]

Frailty was evaluated using the Fried scale, a scale consisting of a list of five criteria. A person was defined as frail if three components, prefrail if two components, were present from the following list: [5]weight loss, endurance, and lower energy as demonstrated by self-reported exhaustion. Low physical activity level: calculated by the physical activity questionnaire, slow walking speed (defined as more than seven seconds to move to a distance of three meters), and low grip strength (defined as the lowest 20%, adjusted for sex and body mass).

#### 2.9.2. Physical Activity Questionnaire [27]

A self-report questionnaire for evaluating physical activity at work, sports, and leisure is based on the Baecke questionnaire for evaluating physical activity [27].

*A medical evaluation* [28] was performed by a physician (anamnesis, a physical examination and collection of laboratory data taken from the HMO). Demographic data, details of diabetes (duration and complications), and A1C values were also collected. A1C is a well-validated measure reflecting mean glucose levels in the 3 preceding months [29]. There is a strong association between A1C values and adverse outcomes in people with diabetes including incident retinopathy, nephropathy, and cardiovascular disease [8]. An association has also been reported between other A1C indices of glucose control and indices of successful/healthy aging such as cognitive function and disability [30–32].

#### 2.9.3. Statistical Analysis

Continuous variables were summarized using means with standard deviations (SD), and binary variables were summarized using counts with percentages. The difference in the distribution of the baseline variables was determined using a chi-square test for counts (percentages), and a *t*-test for means. The association between several physical indices and A1C was assessed using linear regression. The analysis was repeated after adjustment for age and sex.

## 3. Results

153 consecutive individuals over the age of 6o with diabetes who conducted the procedures during evaluation day at the Center for Successful Aging with Diabetes were evaluated. Mean age was 70.7 years, 37.9% were female with a mean of 17.09 years of diabetes and A1C of 7.6%. 37.7% were insulin users, 9.9% experienced an event of severe hypoglycemia, 9.8% were diagnosed as prefrail, and 2.6% were diagnosed as frail. The mean of grip strength, BERG, FSST, 6 min walk test, 30 sec sit to stand test, and TUG were 26.6 (9.3) kg, 52.47 (4.74), 12.16 (4.34) min, 454 (111.7) meters, 11.6 [4], and 8.04 (3.55) sec, respectively (Table 1).

Men compared to women were more likely to be in the highest 6-minute score quartile (vs. lower quartiles). Insulin users and individuals that experienced a severe hypoglycemia episode were less likely to be in the highest 6-minute walk quartile.

People in the highest quartile vs. lower quartiles had more years of education, lower depression scores, higher grip strength, higher score in 30 sec sit to stand test, best results in the balance tests (Berg and FSST), and lower A1C.

There was an inverse association between A1C and score achieved on the 6-minute walk test; with increasing meters walked on the 6-minute walk test, there was a reduction in A1C (*p* = 0.003). There was also an inverse association with the 10-meter walk (*p* = 0.007), BERG balance test (*p* = 0.0006), and timed up and go (*p* = 0.01).

After adjustment for age and sex, only the association between the physical activity score (*p* = 0.005), Berg balance test score (*p* = 0.0176) 6-minute walk test (*p* = 0.0089), 10-meter walk (m/sec) (*p* = 0.0245), and A1C remain significant (Table 2).

**Figure 1:**
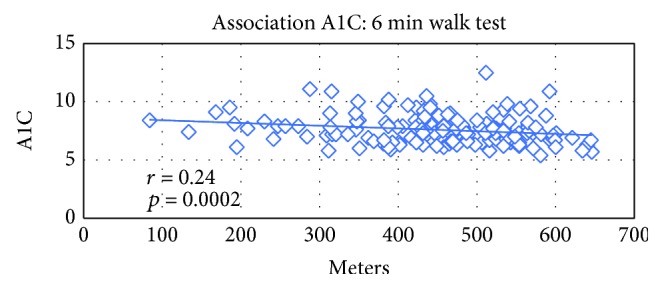
Association between A1C (%) and the 6 min walk test distance (meter).

**Figure 2:**
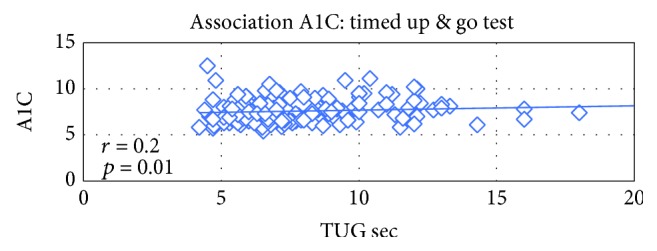
Association between A1C (%) and the TUG test score (sec).

**Figure 3:**
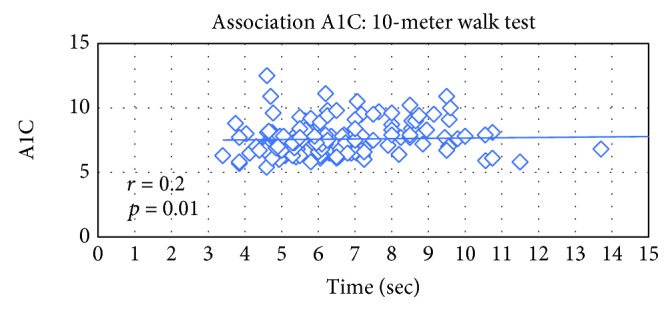
Association between A1C (%) and the 10-meter walk test score (sec).

The table above reveals that significant association between physical indices and A1C was found as seen in the following figures (Figures 1, 2, and 3).

## 4. Discussion

Decreased muscle mass and strength is common in older people in general and in older adults with type 2 diabetes in particular. This study examined the association between glucose control as measured by the A1C measurement and physical performance measures: aerobic capacity, muscle strength, and balance. An inverse association was found between the A1C and the 6-minute walk, physical activity score, the Berg balance test score, timed up and go, and 10-meter walk test. As the older person with diabetes improves the scores on the physical activity questionnaire, the walking distance in 6 minutes, the walking speed of 10 meters, and his balance tests, so does the A1C improve.

Previous studies report similar results. The Baltimore Longitudinal Study of Aging (2003–2011) found that hyperglycemia as expressed by A1C is associated with persistently lower muscle strength with aging [33]and a significant lower handgrip strength compared to normoglycemic controls [10]. In addition, several studies have demonstrated that worse glucose control (i.e., higher glucose values or A1C values) was associated with disability, frailty, and sarcopenia in people with diabetes. For example, among 329 women from the Women's Health and Aging Study II aged 70 to 79, the A1C category at baseline was associated with incidence of walking difficulty and low physical performance [33].

There are several possible explanations for the association between diabetes and sarcopenia. First, it is possible that atherosclerosis disease through coronary artery disease, stroke, and peripheral artery disease causes physical inactivity resulting in sarcopenia and disability. Second, it is possible that diabetic polyneuropathy (DPN), through alterations in the neurotransmission and motor unit remodeling, may be the basis for changes in the motor performance [34].

Finally, advanced glycation end products (AGE) may play a role. AGE formation is a hallmark of type 2 diabetes. AGEs have been hypothesized to play a role in the pathogenesis of sarcopenia through inflammation, through endothelial dysfunction in the microcirculation of the skeletal muscle, and through the cross-linking of collagen in the skeletal muscle [35].

This study has several limitations including its small sample size and the use of a convenience sample of individuals who were either referred by a health care professional or self-referred due to difficulties in managing their disease, thus limiting the ability to generalize these results. Indeed, the relatively low rates of frailty found in this sample demonstrate the selected population included in this analysis. The cross-sectional design limits the ability to determine temporality. Its strengths include the wide range of physical instruments used and the fact that these were conducted by a physiotherapist, a specialist in this kind of assessment.

## 5. Conclusion

In this cohort of older people with diabetes, there was an association between A1C and measures of aerobic capacity and balance. Future prospective analysis of the results from this cohort may allow discretion of temporality. Current guidelines for treating older people with diabetes include recommendations regarding glucose control in older people with diabetes suggesting that level of control should be determined by functional status [36]. Future studies of glucose-lowering medication should include physical and functional assessment.

## Figures and Tables

**Table 1:** Characteristics of study population (*N* = 153).

	6-minute walk test (meter) quartiles								*p*
	≤387.5 (*n* = 38)		>387.5-466 (*n* = 38)		>466-534.5 (*n* = 39)		>534.5 (*n* = 38)		
Gender *N* (%)									
Male	18	(47.4)	16	(42.1)	25	(64.1)	36	(94.7)	<0.0001
Female	20	(52.6)	22	(57.9)	14	(35.9)	2	(5.3)	
Age mean ± SD	73.9	±5.7	71.1	±6.3	69.5	±6.4	68.2	±5.6	0.0004
Education (years) mean ± SD	14.2	±2.8	15.0	±4.1	15.3	±3.1	16.4	±2.4	0.0069
Diabetes duration (years) mean ± SD	22.90	±12.19	17.20	±10.91	12.81	±8.54	16.04	±8.48	0.0164
A1C mean ± SD	7.86	±1.28	7.83	±1.25	7.41	±1.17	7.24	±1.20	0.0699
Insulin *N* (%)	20	(54.1)	14	(37.8)	12	(30.8)	11	(28.9)	0.1002
Severe hypo *N* (%)	8	(22.2)	2	(5.3)	4	(10.3)	1	(2.6)	0.0259
PHQ9 total mean ± SD	6.63	±5.81	4.63	±3.42	3.51	±3.24	3.39	±3.92	0.0196
Prefrail *N* (%)	11	(28.9)	4	(10.5)	0	(0.0)	0	(0.0)	<0.0001
Frail *N* (%)	4	(10.5)	0	(0.0)	0	(0.0)	0	(0.0)	0.0060
Physical activity questionnaire total score mean ± SD	4.52	±2.09	4.81	±1.95	5.67	±1.73	6.68	±1.48	<0.0001
GRIP strength dominant hand (KG) mean ± SD	20.2	±8.1	23.1	±7.6	28.1	±7.3	34.9	±7.4	<0.0001
BERG total score mean ± SD	46.79	±5.89	53.00	±3.24	54.56	±2.34	55.63	±1.05	<0.0001
Time FSST mean ± SD	16.77	±6.39	11.99	±3.27	10.75	±2.01	10.02	±1.56	<0.0001
6MWT (meter) mean ± SD	299.4	±78.4	432.7	±22.2	506.6	±21.1	575.8	±31.2	<0.0001
30 sec sit to stand mean ± SD	8.2	±3.2	11.6	±3.5	12.3	±2.8	14.4	±3.8	<0.0001
TUG (sec) mean ± SD	11.50	±5.20	7.60	±1.71	7.11	±1.68	5.89	±0.86	<0.0001
10MWT (sec) mean ± SD	10.28	±9.26	6.57	±1.25	5.83	±0.93	4.93	±0.73	<0.0001
10MWT speed (m/sec) mean ± SD	1.17	±0.32	1.55	±0.30	1.76	±0.29	2.07	±0.31	<0.0001

**Table 2:** Spearman correlation coefficients between A1C and functional indices.

Variable	*N*	*r*	*p*
Physical activity questionnaire: total score	147	−0.26760	0.0011
GRIP strength dominant hand (kg)	151	−0.11586	0.1566
BERG total score	151	−0.27488	0.0006
Time FSST	137	0.15834	0.0646
6MWT (meter)	150	−0.24142	0.0029
TUG (sec)	151	0.19464	0.0166
30 sec sit to stand	151	−0.04223	0.6066
10MWT (sec)	151	0.20803	0.0104
10MWT speed (m/sec)	151	0.20803	0.0069

## Data Availability

All data from this study are stored and coded together with all data from the study of the Center for Successful Aging with Diabetes at the Sheba Medical Center, Endocrinology Department.
